# Designing for the Co-Use of Consumer Health Technology in Self-Management of Adolescent Overweight and Obesity: Mixed Methods Qualitative Study

**DOI:** 10.2196/18391

**Published:** 2020-06-29

**Authors:** Cynthia M LeRouge, Hyeyoung Hah, Gloria J Deckard, Haoqiang Jiang

**Affiliations:** 1 Department of Information Systems and Business Analytics Florida International University Miami, FL United States; 2 College of Informatics Northern Kentucky University Highland Heights, KY United States

**Keywords:** consumer health technologies, obesity care model, chronic care model, UTAUT, qualitative research, overweight, mobile phone, adolescent, couse, social support, obesity, social influence, consumer health informatics

## Abstract

**Background:**

Overweight and obesity in adolescents has reached epidemic proportions in the United States. Consumer health technology (CHT) can serve as a behavioral and social support tool for the management of overweight in adolescence. Recognizing CHT as a social support tool during design enables input from multiple stakeholders who engage in shared co-use to reinforce and empower adolescents in their self-management efforts.

**Objective:**

This study aimed to explore design requirements and enabling factors for the use of CHT as a social support tool for patients (as primary users) and parents and health care providers (as co-users). Our model incorporates key components of the unified theory of acceptance and use of technology (UTAUT) within the framework of the obesity care model (OCM) by recognizing patient self-management as the central process with the influence of their care support network on CHT use and outcomes.

**Methods:**

This study was part of a larger two-staged usability study combining focus group, semistructured interviews, and usability walkthroughs of CHT mockups from adolescents (BMI in the 85th-99th percentile range), parents, and physicians. In phase 1, 48 adolescents between the ages of 12 and 17 years, 10 of their parents, and 6 health care providers participated in identifying design requirements and enabling factors for the use of a potential CHT. In phase 2, 70 adolescents and 10 health care providers evaluated the CHT mockups and indicated enabling factors and willingness to use the proposed CHT.

**Results:**

Our qualitative analysis identified adolescents’ intention for the use of CHT in alignment with UTAUT elements of performance expectancy, effort expectancy, and facilitating conditions. Our reconceptualization of social influence identified the expectations and envisioned roles of parents and health care providers as co-users and influencing factors on the co-use of CHT in managing overweight in adolescence. Parents were expected to monitor, to provide guidance and motivation, and to suggest modifications in daily habits, for example, recipes and meals, whereas health care providers were expected to encourage and monitor progress in a clinical setting. These expected roles and co-use patterns were congruent among all 3 stakeholders; the co-use of CHT was desired to be minimally invasive for parents and health care providers and controlled by the adolescents.

**Conclusions:**

Our study integrates and extends the perspectives of 2 seminal models to explore design features and social influence roles for the successful user-centered design of CHT for weight self-management in adolescents. Although the co-users (ie, adolescents, parents, health care providers) suggested differing features consistent with their roles, role definitions were congruent. All users recognized the adolescent as the primary user with differential, supportive use from parents and health care providers. This multistakeholder approach can guide successful CHT design that reinforces the collective perspective of self-management.

## Introduction

### Background

The prevalence of childhood overweight and obesity has steadily increased over the past 4 decades in the United States and remains to be high (approximately 1 in 5) [1]. Categorized as weight higher than that considered healthy for a given height, the Centers for Disease Control and Prevention suggest a relative BMI greater than or equal to the 85th percentile as overweight and BMI equal to or greater than the 95th percentile as obese (throughout this paper, to facilitate readability, we will utilize the term overweight as a descriptor for both conditions when the distinction between the 2 conditions is not needed for clarity) [2]. These youths suffer both short- and long-term physical and mental health outcomes [3,4]. Overweight is linked to higher risks for many chronic diseases, including cardiovascular disease, asthma, and type 2 diabetes, which can result in a shorter lifespan [4-7]. Numerous studies have found a strong association between weight status and depression and other mental illnesses [8-10]. In addition, overweight youth are at a greater risk of having obesity and associated health risks as adults [11].

With an already high prevalence of adult obesity in the United States, prevention is a necessary step in reducing both short- and long-term health outcomes, decreasing health care expenditures, and improving quality of life. Recognized as a chronic condition, the etiology of overweight is complex with no definitive cause, although lifestyle behaviors related to food habits, eating patterns, and physical activity are known determinants. Given that life-long habits developed in early childhood and adolescence underlie the foundation for habits in adulthood, early preventative health management is pivotal to addressing the overweight epidemic [12].

Our study recognizes consumer health technology (CHT) as a social tool that enables multistakeholders in the care support network to engage in overweight management efforts by adolescents, thereby reinforcing self-management capabilities. Although previous studies have emphasized the ever-increasing role of patients in the care process, CHT design in the context of a support network has been underexplored [13,14].

### Conceptual Background

#### Consumer Health Technology Role in Support

CHT can play an important role in the self-management of patients with chronic diseases, such as overweight and obesity. Ranging from mobile apps to patient portals, CHTs are broadly defined as information and communication technologies used by consumers for health purposes [15]. The benefits of CHT have been widely discussed with respect to its capability of care transformation, streamlining communication between patients and clinicians, and empowering patients for health decision making. Previous studies highlight that CHT can be integral to enabling (1) a feedback chain (which supports the user in discerning behavioral causes producing either desired or undesired outcomes), (2) information exchange among the user and support network, (3) data management, (4) care planning, and (5) social support from family members [12,13,16-18]. Thus, CHT can provide a means to customize a care regimen by connecting patients to their care team [19-21]. CHT can also function as a decision support tool for both patients and health care providers [21-23]. Tailoring CHT to the preferences and context, for example, the care support network as co-users, can promote the CHT’s potential to reinforce a healthy lifestyle in overweight adolescents [24].

To address a tailored, user-centered approach to deploy CHT, it is important to recognize that overweight management in adolescents is not an isolated health task. Health care self-management in adolescents comprises socially collaborative efforts from adolescents, family members (particularly parents), and care health care providers. Parental influence may be particularly salient because of the sharing of living space, meals, physical activities, and sleeping habits. Overweight adolescents also need guidance and advice from health care providers who can design an individualized care plan for the eating patterns and levels of physical activities of adolescents. Given their integral yet differential roles for influence and support, parents and health care providers may influence how adolescents use CHT.

#### Caregiver Role in Support

**Figure 1:**
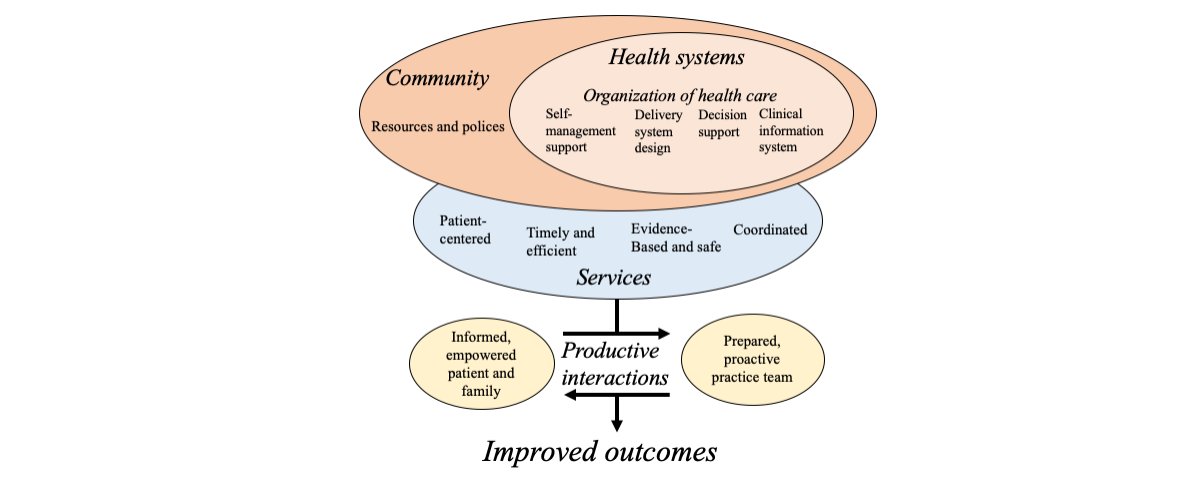
Chronic care model.

Effective prevention and care of chronic illness require an organized delivery system linked with multiple supportive community resources [25]. Such support systems are not generally recognized nor the needs of those with chronic illness adequately met in a traditional primary, acute health care system [26]. The chronic care model (CCM) addresses these complementary systems (Figure 1) and provides an approach to high-quality chronic care characterized by productive interactions between health system health care providers and patients that consistently provide assessments, support for self-management, optimization of therapy, and follow-up (Figure 1) [27].

**Figure 2:**
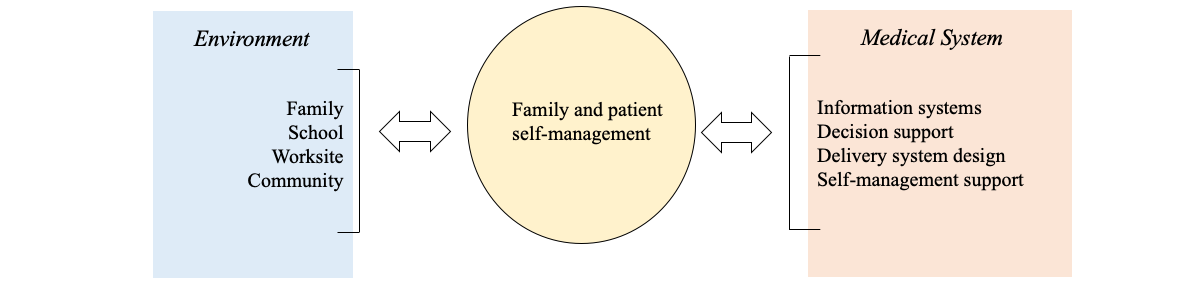
Obesity care model.

Development of the CCM was based on evidence from system interventions to improve care for various chronically ill populations, and subsequent implementation generally focused on changes within care delivery organizations [28,29]. To provide more focus on the environments outside the clinic or health system walls, the obesity care model (OCM) modifies the original CCM framework to place equal emphasis on the community and the medical system (Figure 2) [30]. The OCM places patient and family self-management as the central process to improve weight management. As shown in Figure 2, the role of health care health care providers to assist in self-management includes information systems and decision support, which may be implemented through CHT [31].

The CCM and OCM provide perspectives and structures to investigate the multilateral roles adolescents, parents, the community, and health care providers play in managing the adolescent’s weight. These critical players may also play an important role in the success of CHT as secondary users or co-users. CHT can streamline shared care tasks among patients, parents, and health care providers. In translating OCM or CCM concepts to CHT, members of the adolescent’s care support network (eg, parents and health care providers) may act as co-users by noting goal accomplishments or sharing information (eg, motivating messages); health care providers can provide feedback and support as well as contribute to the care plan.

#### Recognizing the Co-User Role in Adoption and Use

The phenomena of a care support network in relation to CHT and co-users of technology can be theoretically linked to the notion of social influence referenced in the unified theory of acceptance and use of technology (UTAUT) [32]. The UTAUT identifies 4 determinants of attitude and intent to use information technology: (1) perceived usefulness (performance expectancy), (2) effort expectancy (ease of use), (3) social influence (significant others’ evaluation of technology use), and (4) facilitating conditions (supporting infrastructure for technology use). These UTAUT determinants help us explain technology adoption behaviors by health care consumers and health care providers [33-38]. Understanding, contextualizing, and reacting to the precursors of *intent to use* by the primary technology user (ie, adolescent) is a critical foundation for the design and adoption of CHT. Extending this understanding to supportive co-users (ie, secondary users) in alignment with OCM or CCM concepts can improve CHT design and increase the understanding of the contribution of the co-user roles.

**Figure 3:**
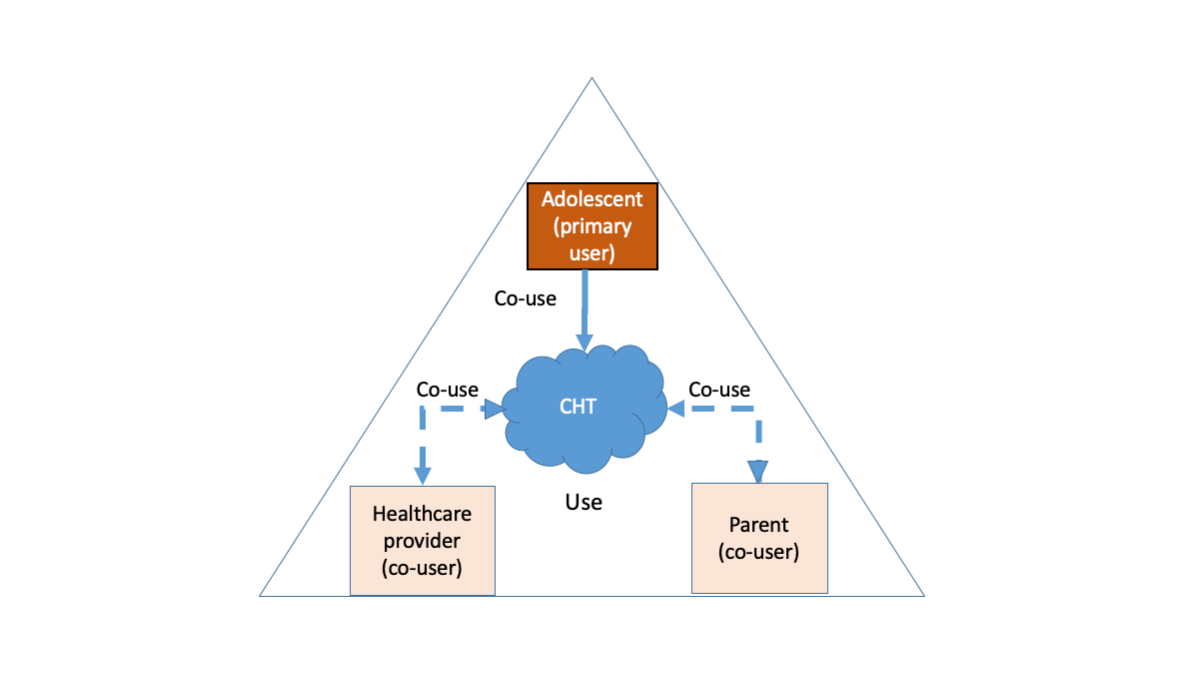
Design perspective of primary and co-user roles in consumer health technology.

The UTAUT social influence antecedent recognizes that an individual’s intention to use technology may adjust or modify their technology adoption behavior (a concept comparable with the environmental influences found in the OCM). The care support environments referenced in the OCM may provide social influence (particularly through indirect or direct engagement with the CHT that may impact the primary user’s opinions or behaviors toward using the CHT and self-management) to further explore the possibilities of CHT to assist adolescents’ self-management efforts (Figure 3). Research shows that social influence impacts dietary behavior and adolescents’ readiness toward their chronic care health [39,40]. Unfortunately, there is a lack of literature that integrates the co-user’s perspectives and expectations regarding the intention to adopt CHT for adolescent overweight management.

### Goal of This Study

**Figure 4:**
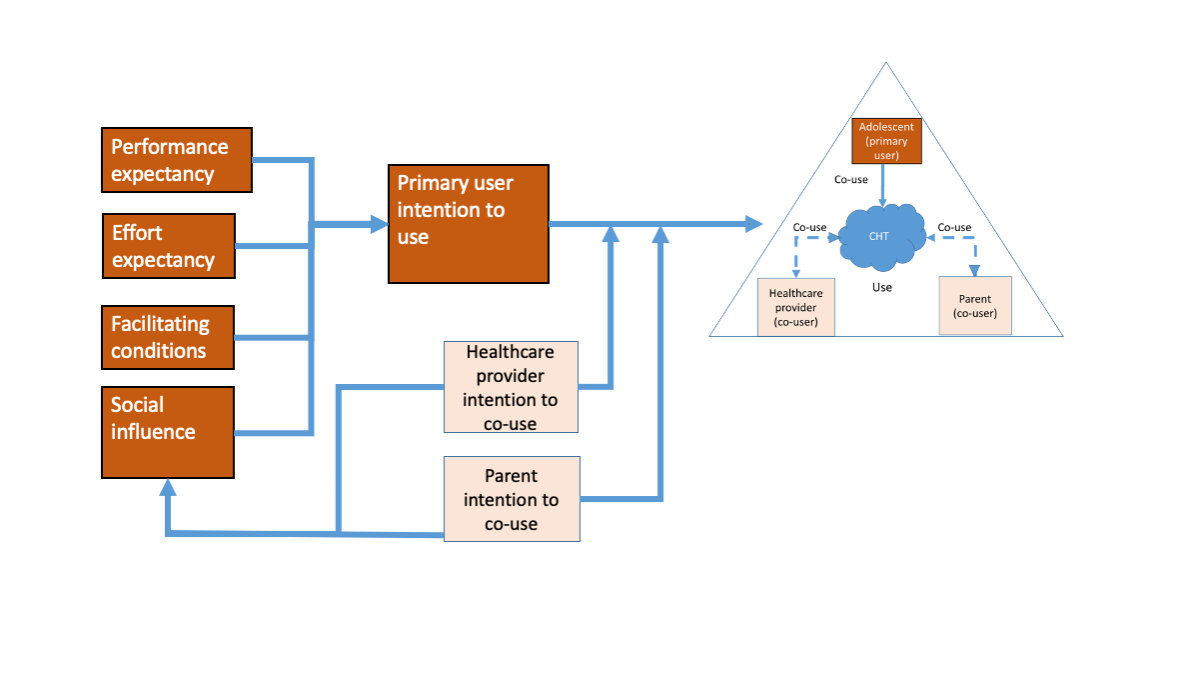
Intention to co-use model. CHT: consumer health technology.

Our research model presented in Figure 4 utilizes the UTAUT to identify the impact of social influence on the co-use intention for adolescents, parents, and health care providers. The model also incorporates the OCM by recognizing the use, support, and influence of the primary CHT user (the adolescent) by parents and health care providers. That is, a patient’s CHT use behavior can be influenced not only by the extent to which an individual believes how others view his or her use of technology but also by the way others in the patient’s support network and secondary users assess the technology and the potential benefit of the patient’s technology utilization. Our research model simultaneously incorporates the factors that shape a primary user’s CHT adoption and that of secondary users.

In this model, we reconceptualize the social influence construct that can connect the adoption of 3 distinct users (patient, provider, and caregivers, including parents) in the same nomological care network. The social influence construct takes the primary users’ perception of the use and role of co-users into account when explaining the primary users’ information technology adoption intention. We specify social influence for our context of interest as the way an adolescent perceives the role of others in the OCM (eg, health care providers, community including peers, and family) for their use in CHT apps.

We apply this model to a qualitative study of CHT design in the context of a support network for overweight and obesity in adolescents by first contextualizing the UTAUT constructs of performance expectancy, effort expectancy, and facilitating conditions as antecedents of use of CHT for adolescent overweight and obesity by the primary user (adolescent). We will then reconceptualize social influence in relation to the co-use of CHT in the social cycle of adolescent-parent-health care providers supporting the management of adolescent overweight and obesity. More specifically, we examine how multiple users perceive their shared care tasks that can further lead to their envisaged co-use intention. This study’s unique attention to each user’s perception and expected design requirements for CHT prototypes provides an in-depth understanding of feature-based CHT design linking to intention to use CHT from multiusers.

## Methods

### Methodology

This qualitative study integrates the UTAUT framework within the OCM context for the management of overweight in adolescents. Through this integration of support and use concepts, we leverage a user-centered design methodology to examine users’ adoption of CHT for overweight management in adolescents.

This paper is part of a more expansive user-centered design research project that explored questions and issues related to requirements analysis, human-computer interaction, prototyping, usability, technology evaluation, and technology adoption by overweight adolescents, their caregivers, and their health care providers regarding technology to support self-management and caregiver connections. This design project included 2 phases: (1) analysis of needs, preferences, and human-computer interaction in social context and (2) design, prototyping, and usability. We received an institutional review board approval from Saint Louis University. Here, we focus on aspects of the methods, particularly related to the research intentions of this study (further details of the global study are reported elsewhere) [41].

To demonstrate rigor with respect to study validity, we carefully considered the research methods used (ascribed to user-centered design approaches), the participants (sources of data), and the number of participants. For qualitative studies, the sample size may be considered a matter of judgment and experience in evaluating the quality of the information collected against the uses to which it will be put, the particular research method, and purposeful sampling strategy that focuses on individuals with the experience to provide appropriate insights for the targeted population [42]. A study of sample size for qualitative research by Faulkner found that the exact numbers required for usability tests were not as important as the participants representing the target population [43]. For each stage and with each method of data collection, selection of our participants was purposeful to include individuals with direct knowledge of the points of inquiry to accurately reflect the targeted user population (ie, overweight or obese adolescents) [42]. We balanced the purposeful determination of appropriate informants (to share insights on the adolescent as primary use and parents and health care providers as secondary users) with heuristics on sample size and the overall need to achieve saturation in identifying general goals for data collection and points of saturation. Saturation is defined as the point at which additional data collection no longer generates a new understanding [44]. For qualitative research in general, a recent review showed that the median sample size was between 28 and 31 to achieve data saturation [45]. In total, this study included 143 individuals, far exceeding this norm. However, this number should be interpreted with some caveats. The sample size in qualitative research may refer to the numbers of persons (say for individual interviews or individual thinking out loud activities as a usability exercise) but also to the numbers of interviews (such as a focus group), given the different units of analysis [42]. We provide further details, as appropriate, in each phase to further showcase rigor.

#### Phase 1

The goal of phase 1, with respect to the scope of this study, was to detect desired application areas to assist with self-management and to identify design and functional requirements as a means to inform design details to meet the adolescents’ performance and effort expectancy (UTAUT model). It was also important to determine the context of self-management (facilitating conditions from UTAUT and OCM) for these adolescents. In addition, this phase began to explore the elements of productive interactions (CCM model) among adolescents, their families, and health care providers to their self-management.

Phase 1 techniques included 10 one-hour user-driven focus groups with adolescents participating in 2 different sessions in an accredited weight self-management program in the Midwest, United States. The weight loss camp allowed the researchers to access individuals with relevant experience and appropriate insights on the usability of apps for weight self-management by adolescents. The summer camp program (specifically, Camp Jump Start, a recognized adolescent weight loss and healthy living summer camp program, grounded in evidence-based techniques and led by a health careprovider) attracts clients from across the United States. The study participants included a diverse group of black, white, and Hispanic adolescents, aged between 12 and 17 years, from various socioeconomic backgrounds. Both males and females were included in the study. The inclusion criteria were based on age, computer use, and BMI (participants were in the 85th-99th percentile range). Each focus group had 3 to 7 participants, and the total number of participants for the focus groups was 48. Quantitatively, it is recommended to have 3 to 5 groups per project to reach saturation [46]. Further, the general recommendation is that each group should have a minimum of either 3 to 4 and a maximum of 12 participants [46,47].

Then, 15 semistructured interviews with the parents of adolescents participating in the summer camp and 6 semistructured interviews with pediatric physicians of these children were conducted. Selected focus group and semistructured interview questions were developed with consideration of the UTAUT, CCM, and OCM factors [27,30,32]. The interviews were recorded over the phone before adolescents completed their programs.

The multiple focus groups and interviews allowed the researchers to continuously analyze the content of the transcripts to identify themes to the saturation point, that is, the point at which additional data collection no longer generates new information or understanding [48]. Although content-coding and analysis appeared to reach saturation following the seventh focus group, the final 3 focus groups confirmed this perception. Similarly, saturation was reached for the purposes of this phase with less than the actual number of interviews conducted for both the parent and health care provider interviews. The focus groups and interviews beyond saturation helped to validate the identified themes.

Details regarding application areas identified from this phase (social networking, motivation, cooking, physical activity management, and food management) and insights regarding the contexts of use (environment and interactions) were used to develop a series of screen mockups and usability study protocols that were used in phase 2.

#### Phase 2

As with phase 1, the selection of our participants in phase 2 was purposeful to accurately reflect the targeted user population (ie, adolescents who were overweight or obese and health care providers with practices that regularly serve patients with this condition) [42]. Again, we exercised rigor to ensure internal and external validity by extending the number of participants beyond the typical range of 5 to 20 participants [43]. As with the focus groups, we found that data from the number of participants consulted exceeded that which was necessary to reach a saturation point and provide a comfortable level of confidence regarding internal and external validity.

We recruited 70 adolescents attending the weight management camp session to participate in usability sessions that lasted approximately 1 hour. Study participants in this phase of the research shared the demographic and inclusion criteria applied in phase 1, reflecting the targeted population of users. The adolescents first answered some general background questions (ie, age, technology access questions, current methods of self-management), then performed a series of usability tasks related to the mockups that helped to inform effort and performance expectancy (UTAUT), and addressed questions regarding their vision of context of use and co-users (informed facilitating conditions and social influence).

In the final step of phase 2, we conducted 10 interviews with health care providers (pediatricians and family practitioners) over the telephone. We selected pediatricians and family practitioners using the snowball sampling method and received their consent before arranging our interview sessions, which lasted between 45 and 60 min. These health care providers were experienced in treating our target population of overweight and obese adolescents.

The interview protocol focused on the general reactions to the screen prototypes. The pediatricians and family practitioners shared their thoughts about challenges and barriers related to patient utilization of technology, their personal assessments of the prototypes, reactions to some key insights from the adolescents’ usability assessment, and perceptions about their role in co-use (including the integration of CHT summative patient reports in these health care providers’ practice).

### Data Analysis

Phase 1 and 2 interviews, focus groups, and usability walkthroughs were audio-recorded, transcribed, and reviewed for transcription errors. We conducted a data analysis of all transcripts using Dedoose, a qualitative data analysis tool for data codification, classification, and treatment. Guiding principles proposed by Lee and Baskerville [49] were applied to develop understanding and insights from collected data.

The analysis consisted of 3 stages. First, 2 research team members independently coded text to the constructs in our intention to use the co-use model (Figure 4) to extract some general impressions of model fit and application to adolescent overweight. Second, the coders added child codes (subthemes) to afford more detailed levels of explanation of each of the constructs in the integrated UTAUT and OCM or CCM models to further understand the nature of each of these constructs in the context of the study. Third, another research team member independently reviewed all codes (100% code review) to determine the propriety of the assigned codes to the construct of interest, noting potential issues and questions to the coding of the transcripts. Overall, the team met regularly during the coding process to iteratively discuss initial coding, refine coding categories, and reach consensus regarding identified subthemes [50]. Reconciling issues were minor and primarily consisted of agreeing on combining child codes, appropriate titles for code names, and addressing coder thoughts on whether a new code was required. Data analysis was complete after the team reached a consensus that all quotes were appropriately coded, and the resulting themes under each construct were stable [51].

## Results

### Overview

Our investigation underscores 2 different aspects of adolescents’ CHT use: primary use and co-use with other members of their care support team. That is, adolescents act both as primary users and co-users, with parents and health care providers participating as secondary users in supportive roles consistent with the environment and supportive concepts of the OCM. In alignment with our model, we leverage constructs from the UTAUT model that serve as antecedents to intention to use (performance expectancy, effort expectancy, facilitating conditions, and social influence) [32]. The first 3 constructs are closely linked to the primary (sole) use of CHT, whereas social influence demonstrates the extent to which adolescents desire co-use of CHT with other key users (eg, parents and health care providers). Underpinning co-use, as part of social influence, are the adolescent’s expectations of desirable inputs and feedback from co-users.

In the following section, we first link the UTAUT constructs of performance expectancy, effort expectancy, and facilitating conditions to adolescents’ perceptions of the primary use of CHT. Then, we examine the perceptions of the role of co-users as social influence that may alter adolescents’ intention to act as primary users and lead to the co-use of CHT with parents and health care providers**.**

### Performance Expectancy

In our research context, performance expectancy is viewed as the degree to which an adolescent believes that the availability and use of CHT functions and features may help him or her with weight management. Our results indicate that adolescents anticipate that a successful CHT app would promote, reinforce, or extend their motivation to combat obesity, try healthy food alternatives, and exhibit healthy behaviors. Viewing CHT as motivational support to their busy schedules between school, extracurricular activities, and social obligations, they identified a list of key features related to behavioral awareness and guidance and education on healthy living. In essence, features that would provide them with tools that underpin and promote self-management.

CHT features identified by the adolescents related to behavioral awareness and guidance included (1) timely reminders and automated notifications to log in and complete information (eg, food diary entries), (2) prompts to take certain actions (complete a physical activity), and (3) educational information on healthy food and living choices. To assist in learning healthy behaviors and altering lifestyle habits, adolescents suggested CHT features that would (1) set personal goals, (2) track their daily behaviors (eg, caloric intake and calorie burning), and (3) provide rewards (eg, awards, coupons, or discounts for healthy foods at stores or restaurants). They also expressed a desire to keep all of the tracking information and materials in one location on a computer or smartphone. To keep track of their progress, they were interested in visualizing graphs of their weight loss efforts.

### Effort Expectancy

We regard effort expectancy as the amount of effort adolescents are willing to exert to use CHT in their self-management of overweight. Our interviews revealed that adolescents expect ease of use of the system interface as one of the most important factors for the adoption of CHT. Adolescents mentioned that minimal effort was made possible by an uncomplicated interface to easily find and add their own health information. To reduce writing or typing efforts, features such as drag-and-drop capabilities, drop-down menus, and search function were mentioned as means to ease efforts to find, add, and edit personal information. Furthermore, adolescents desired the system to display their progress and history readily without much effort.

In addition, they wanted CHT apps to be flexible, for instance, to allow infrequent data entry, smooth integration among its modules, and connections to other external apps. The adolescents also desired the capability to edit app components such as recipes so that they could build or edit recipes to fit their own tastes.

Simplicity in use was particularly important for smartphone interfaces, as some adolescents indicated that they would like some level of access *on the go* via smartphones, given the smaller screen size of the phone.

Finally, the adolescents suggested an entertainment component, such as games and avatars, to be featured in the CHT app. They indicated that game features, along with the ability to share game progress, would motivate them to return to the app and reduce their perceived effort to use.

### Facilitating Conditions

Facilitating conditions reference a belief by the adolescents that certain technical and contextual conditions support their use of the CHT app. The roots of this UTAUT construct include perceived behavioral control, facilitating environmental conditions, and compatibility. Our results show that adolescents deemed compatibility as a major factor impacting their use of the CHT. Adolescents indicated that access to the CHT app from multiple platforms or devices such as phones, tablets, and computers would enable them to track progress more easily. The most preferred platform was smartphones, as most adolescents stated that being able to track while *on the go* would support their frequent usage of the app. Lack of advertisements was also mentioned as a facilitating condition for using the apps.

The adolescents identified a range of conditions that could be viewed as nonfacilitating, particularly technical issues that could prevent their use of the CHT. These potential barriers included the cost of the app, password memorization, and system crashes. In addition, connectivity issues associated with Wi-Fi or the internet could block CHT use.

Overall, overweight adolescents view CHT apps as motivational and capable of altering behavioral patterns. Our analysis of primary user perceptions for intent to use a CHT identify key features and factors compatible with their day-to-day use for self-management. Acceptance and use of the CHT depend on the incorporation of features noted above into the design of the app.

We now turn to our attention to perceptions of the primary user (overweight adolescents) and the supporting community of parents and health care providers regarding the social influence and co-use of CHT.

### Social Influence as Co-Use of Consumer Health Technology

In our research context, we integrate the UTAUT concept of social influence that focuses on individuals’ perceptions that the people close to them believe that they should try using the technology with the OCM, which considers patient and family engagement and empowerment. Our conceptualization examines the degree to which an adolescent perceives the role of others in the OCM, for example, parents and health care providers, and their co-use of CHT in their self-management of overweight. We also explore the perceptions of co-use by the health care providers and parents (the OCM support network) of these adolescents regarding their role and use of the CHT.

#### Social Influence and Co-Use: Adolescents’ View

Adolescents identified key participants in their community of support and social influence as family, including parents and siblings, health care providers and peers or friends, and nominated parents and health care providers as co-users of the CHT app in their weight loss management program. They viewed the role of social influence to include acknowledging challenges, setting up motivational tasks, respecting inputs, and sharing achievements.

By sharing with others through co-use of CHT, the adolescents thought they could discuss difficulties and achievements, mindfully monitor their progress, and receive encouragement from their support community in a timely fashion. An exemplary quote summarizing these thoughts states:

You could talk to other people about your difficulties and they could give you tips and advice and just help each other out.

Simultaneously, however, adolescents draw a clear line regarding the boundaries of co-use in terms of data, information, and management. Self-management should be guarded so as not to give up control. Others participating in cousage should not be able to modify the information or alter the adolescents’ weight loss process, for example:

I wouldn’t want them to like go in and mess everything up.

##### Adolescents’ View of Co-Use by Family Members

Although adolescents acknowledged multiple family members as potential co-users, they clearly identified parents as one of the major sources of social influence. They indicated that parents could provide support by reviewing progress, providing encouragement, cocreating information content, for example, recipes, and encouraging family interaction. Adolescents also mentioned creating a competition between family members—each setting up weekly goals and seeing who does best.

Generally, parents as co-users of the app would demonstrate engagement while empowering adolescents’ self-management and drive toward achieving their goals, providing motivational support, and adding accountability by reviewing their progress.

##### Adolescents’ View of Co-Use by Health Care Providers

The adolescents viewed the role of health care providers’ social influence as creating the obesity care plan and encouraging through monitoring progress, providing feedback, and acknowledging achievements. Having the guidance and support of their health care provider outside of visits to physicians provides adolescents with confidence during different steps of their behavioral modification process.

Our results show that adolescents confirm the importance of and need for social influence and a supportive community from parents and health care providers to provide guidance and assistance with their self-management of overweight. However, this influence has clear boundaries and should be supportive and empowering, not interfering. Defining who can help them and how others can help them within their desired social influence is instrumental to a successfully designed CHT that all users (primary and co-users) will engage with as intended.

#### Social Influence and Co-Use Intention: Parents and Health Care Providers

The perceptions of co-users regarding their role and use of CHT are a critical, yet often overlooked, component for both design and use. Here, we report the perceptions of 2 key co-users, parents and health care providers, whose social influence on overweight adolescents is an essential component of our integrated conceptualization of the concept.

##### Parents’ View of Co-Use

Our interviews with parents revealed their willingness to support their children and to participate as co-users of CHT designed to assist their self-management of overweight. Parents identified forms of social influence and support and desirable characteristics of the CHT.

First, parents clearly recognized the adolescent as the primary user of the CHT and themselves as a secondary user. Although wanting to provide support in a number of ways, they acknowledged the need to respect the privacy of their children. Parents expressed these perceptions through statements such as the following:

I can see myself monitoring, as a kind of oversight... But if you get too involved, then it’s no longer interesting to them... they feel like it’s invading their privacy.

To assure the adolescents of their privacy, parents indicated that having their own log-in was important whereas tying specific aspects of the CHT together for input and support:

…you’re probably more successful together. You have someone to talk with and give you support.

Parents defined their support role as one to provide guidance, shared accountability, and motivation:

I could see myself being more involved, you know, like a participative role where maybe he's coming up with something and I'm giving him feedback.

Yet doing so, only within the areas sanctioned by the adolescent as indicated by this sentiment:

I think there’s a part of me who would really like to peek over her shoulder and see everything she’s doing, but I also feel like she deserves her privacy, and she deserves that respect if she wouldn’t want to share it... I think I would really want to go just based on her comfort level.

The value of the CHT itself for motivation and guidance was equally recognized by parents:

I think it could be helpful if it could keep her motivated.

Regarding features that would support their co-use, the parents suggested the technology to be general enough to monitor the progress of their children and allow them to provide timely advice or input without being invasive. One thought expressed was to provide icons such as a “yellow happy face” to provide encouragement.

Parents are clearly willing to provide their children with support to utilize CHT tools in an everyday setting and to provide support through the co-use of the CHT. Interestingly, parents distinguished themselves as secondary users in supportive roles that encouraged their children’s self-management as primary users of the CHT.

##### Health Care Providers’ View of Co-Use

Health care providers perceived themselves as a source of encouragement and support in adolescents’ obesity management. Health care providers acknowledged their role in providing medical system support to go beyond office visits and referenced the need for a means to acknowledge adolescents’ progress and provide feedback on a regular basis. Given that health care providers manage a number of adolescents with obesity, they readily acknowledged that their ability to communicate and extend this support between office visits could be streamlined by the co-use of CHT.

Due to daily time constraints in physicians’ offices, health care providers expressed their strong interest in giving encouragement by using the automatic CHT system features to quickly send their rewards, notes, or feedback that “wouldn’t be hard and wouldn’t take a lot of time.” Minimizing time and effort is critical for health care providers’ co-use of the CHT application.

In addition, health care providers wanted the CHT to track adolescents’ progress and provide a quick summary report showing how their patients are progressing toward their individual goals. The health care providers also indicated that they would value an alerting feature that noted both achievement and lack of progress or lack of data input. Alerts would allow a real-time response and signal the provider to contribute appropriate, timely feedback through the CHT to the adolescent.

Overall, health care providers are inclined to encourage adolescents’ primary usage of the CHT and want the ability to reward their achievements or encourage progress through efficient co-use capabilities. As health care providers’ time is limited and they may manage multiple primary users (patients), effective co-use relies on automatic features to monitor their patients’ daily progress, provide weekly reports, and efficient means for providing support to adolescents for obesity management.

## Discussion

### Summary of Insights

This paper examines the factors influencing the intent to use CHT in the management of overweight by adolescents and the role of social influence from the perspective of adolescents, parents, and health care providers. Social influence cannot only impact the use of CHT as conceptualized by the UTAUT model but also provide meaningful engagement of parents and health care providers in a way that empowers adolescents’ self-management of overweight.

Through the inclusion of both primary users (adolescents) and co-users (parents and health care providers) in our study, we can contrast perspectives to assess the congruence in the defining primary user and co-user roles for CHT in the self-management of adolescent overweight. Recognition of the adolescent as the primary CHT user is consistent for all 3 contributors. Both parents and health care providers view their roles as secondary or co-users and are responsible for providing information, feedback, encouragement, and support. Differences in the roles of these secondary users are apparent, as health care providers are part of the medical system, whereas parents are part of the environment of family and community support. Overall, health care providers were viewed in the expert role providing plans and directives along with motivational feedback, whereas parents were seen as critical in supporting, encouraging, and motivating adolescents in the process of self-management. Rather than interfering, the roles of both co-users were directed toward empowering adolescents. The medical system is more independent from the primary user and assumes more oversight of the adolescent’s progress, albeit through automated CHT features. Parents are more interdependent; however, shared access is controlled by adolescents in recognition of the need for privacy and respect.

Our findings point to the importance of the visibility and transparency of co-use in the realm of CHT. In our study, adolescents would choose the activities and inputs visible on the CHT to specific co-users, possibly with some sensitive information hidden from parents but available to health care providers. However, the activities of both parents and health care providers as co-users are to be visible to their children to create a noninvasive, shared sense of accountability for obesity management.

By explicitly looking for role congruence regarding social influence from parents and health care providers, we found that as co-users, both parents and health care providers are willing to engage in the process and fulfill their role to empower the primary user consistent with the OCM. These findings correspond to the existing studies that highlight the needs of including parents and physicians in the process of mobile health (mHealth) app design and implementation to promote the use of mHealth technologies [52-57].

From an adolescent’s perspective of CHT use in overweight self-management, our results establish that adolescents are willing to (1) use CHT features that promote behavioral awareness and guidance, (2) input and monitor progress toward weight loss goals across platforms, and (3) allow motivational inputs and encouragement (social influence and support) from parents and health care providers as co-users within defined areas that respect their privacy. As co-users of the CHT, parents and health care providers recognize that adolescents want respect for their privacy whereas desiring feedback to motivate and inspire. The provider sought independent and automated feedback features whereas parents desired to project shared accountability, pulling together to motivate achievement of goals and encouraging progress. We invite future research to explore the need and benefits of sustained use for primary users and co-users.

When designing CHT for obesity management, incorporating the perspectives of the OCM into UTAUT can provide a broader, more comprehensive view of social influence—one that provides input from patients as primary users, parents as part of the supportive environment, and health care providers as part of the medical system. Responding to each of these groups, their concern for CHT features and the role they play can improve successful design and adoption.

### Contributions of the Study

The contributions of this paper are three-fold. First, we investigate the construct of the social influence of the UTAUT framework through the lens of the OCM and incorporate perspectives of 3 users. We expand the social influence construct by exploring the primary user’s specific expectations of secondary users, namely family members (particularly parents) and health care providers, for social support as an antecedent to use CHT to manage adolescent overweight. Our findings point to the dynamic nature of social influence among adolescents, parents, and health care providers. Perceptions of the varying roles demonstrate congruence among the users; this common recognition of adolescents as independent, primary users may empower and encourage their ongoing use of CHT in the self-management process.

Second, we propose the role of CHT co-use for overweight to be tailored to the OCM framework that extends the intention to use construct to recognize CHT co-use (intended behaviors for use) by family members (particularly parents) and health care providers as secondary users that simultaneously influence the adoption of CHT for managing overweight in adolescents. Acknowledging the importance of designing a user interface that is appealing to the users and responds to their needs is central in developing user-centered CHT systems [51-57]. This study’s approach to the design of CHT allows for simultaneous use in the management of adolescent obesity across 3 system users. We found that in the OCM context, the co-use of CHT may support parents and health care providers as supportive social influencers in adolescents’ overweight management process while empowering adolescents in the leading role. To the best of our knowledge, this is one of the earliest studies to consider co-use in a chronic care partnership with adolescents. Such a co-use care partnership should be extended with a series of further studies.

Finally, our user-centered design approach identifies envisioned features that directly influence intention and use for 3 co-users. The design requirements revealed from all 3 user groups can further lay a groundwork to tailor and contextualize the features of CHT systems in the chronic care management context.

With the growing role of CHT in the treatment of chronic care, our study has many practical implications for enhancing active co-use from patients, parents, and health care providers. Our study suggests that CHT vendors provide a user-centered design for multistakeholders to manage *shared* health care tasks for active participation and effectiveness in self-management. To better promote a broader range of CHT usability, vendors and developers need to relate to co-users and accommodate the intended users’ needs and requirements.

Our study may also inform health system health care providers who need to focus on the impacts of coengagement from parents and care health care providers in special care task contexts. Parents generally influence the lifestyle habits and decision making of children and adolescents. Therefore, understanding the co-use of CHT allows for articulation of the roles of parents (or caregivers) and care health care providers to provide tailored CCM and OCM plans for various care contexts.

### Limitations

As with most qualitative studies, a generalization of the results must be approached with some caution. Although the study included a diverse set of adolescent participants, the study involved a limited sample of adolescents between 12 and 17 years. Our findings may not be applicable to children younger than 12 years of age or older than 17 years. First, the study also included a limited sample of parents of these adolescents and health care providers serving the adolescent population. Second, our participants were self-selected to participate in the weight management programs, indicating their motivation and intention (and by association, perhaps their parents’ willingness to provide support) was higher than average. Thus, our results might be skewed toward highly motivated adolescents who are willing to use and co-use CHT with their parents and health care providers. Finally, our study does not include all possible members of a social support network (eg, peers and other family members) that may fill or contribute to the co-use roles we have outlined or introduce additional co-use possibilities. Each of these limitations is a call for future research to extend the scope of this study.

### Conclusions

Social influences, as envisioned in our study (ie, the integration of the UTAUT focus on perceptions of others with the OCM’s consideration of patient and family engagement and empowerment) can affect the use of CHT as part of weight loss self-management. Our study investigated the desired CHT design features and co-user roles that can support not only the primary user but also the support environment (through parent co-use) and medical system support (through provider co-use) in self-management of adolescent overweight.

This study confirmed the wide applicability of the UTAUT model in a consumer health informatics context while recognizing the importance of co-use in the environmental and medical system components of the OCM. We found a strong willingness among adolescents to adopt co-use CHT features that assist with motivation, monitoring, and rewarding features in conjunction with parents’ and health care providers’ participation. Furthermore, we demonstrate that the expected roles and requirements of CHT co-use correspond to each other’s needs in the care model, whereas differences in the way CHT is co-used is transparent.

The study established differing features to encourage intention of use by each of the co-users but congruence in defining the primary and co-users’ roles. All co-users recognized the adolescent as the primary user with control of input and information in concert with concerns for privacy.

Overall, this study promotes awareness that the design of CHT can link multiple users to fulfill their roles in the management of adolescent overweight through complementary and shared care tasks. The results of this study should prompt researchers and developers to recognize that CHT focused on adolescent overweight and obesity, and arguably many CHTs targeted to support the management of chronic conditions, should be designed, developed, and tested with the awareness of co-use to reinforce the social influences required for effective self-management.

## Abbreviations

CCM: chronic care model
CHT: consumer health technology
mHealth: mobile health
OCM: obesity care model
UTAUT: unified theory of acceptance and use of technology

## References

[1] Skinner AC, Ravanbakht SN, Skelton JA, Perrin EM, Armstrong SC (2018). Prevalence of obesity and severe obesity in US children. Pediatrics.

[2] Centers for Disease Control and Prevention.

[3] Reilly JJ, Methven E, McDowell ZC, Hacking B, Alexander D, Stewart L, Kelnar CJ (2003). Health consequences of obesity. Arch Dis Child.

[4] Reilly JJ, Kelly J (2011). Long-term impact of overweight and obesity in childhood and adolescence on morbidity and premature mortality in adulthood: systematic review. Int J Obes.

[5] Dietz WH (1998). Health consequences of obesity in youth: childhood predictors of adult disease. Pediatrics.

[6] Park M, Falconer C, Viner R, Kinra S (2012). The impact of childhood obesity on morbidity and mortality in adulthood: a systematic review. Obes Rev.

[7] Mohanan S, Tapp H, McWilliams A, Dulin M (2014). Obesity and asthma: pathophysiology and implications for diagnosis and management in primary care. Exp Biol Med (Maywood).

[8] Russell-Mayhew S, McVey G, Bardick A, Ireland A (2012). Mental health, wellness, and childhood overweight/obesity. J Obes.

[9] Luppino FS, de Wit LM, Bouvy PF, Stijnen T, Cuijpers P, Penninx BW, Zitman FG (2010). Overweight, obesity, and depression: a systematic review and meta-analysis of longitudinal studies. Arch Gen Psychiatry.

[10] Morrison KM, Shin S, Tarnopolsky M, Taylor VH (2015). Association of depression & health related quality of life with body composition in children and youth with obesity. J Affect Disord.

[11] Singh A, Mulder C, Twisk J, van Mechelen W, Chinapaw M (2008). Tracking of childhood overweight into adulthood: a systematic review of the literature. Obes Rev.

[12] Steinberg L (2001). We know some things: parent-adolescent relationships in retrospect and prospect. J Res Adolesc.

[13] Kaplan B, Brennan PF (2001). Consumer informatics supporting patients as co-producers of quality. J Am Med Inform Assoc.

[14] Novak L, Unertl K, Holden R (2016). Realizing the potential of patient engagement: designing IT to support health in everyday life. Stud Health Technol Inform.

[15] LeRouge C, van Slyke C, Seale D, Wright K (2014). Baby boomers' adoption of consumer health technologies: survey on readiness and barriers. J Med Internet Res.

[16] Cramm J, Nieboer A, Finkenflügel H, Lorenzo T (2013). Disabled youth in South Africa: barriers to education. Int J Disabil Hum Dev.

[17] Drawz P, Rosenberg ME (2013). Slowing progression of chronic kidney disease. Kidney Int Suppl (2011).

[18] Shegog R, Bamps YA, Patel A, Kakacek J, Escoffery C, Johnson EK, Ilozumba UO (2013). Managing epilepsy well: emerging e-tools for epilepsy self-management. Epilepsy Behav.

[19] Cygan H, Reed M, Lui K, Mullen M (2018). The chronic care model to improve management of childhood obesity. Clin Pediatr (Phila).

[20] Fico G, Arredondo M (2015). Use of an Holistic Approach for Effective Adoption of User-Centred-design Techniques in Diabetes Disease Management: Experiences in User Need Elicitation. 37th Annual International Conference of the IEEE Engineering in Medicine and Biology Society.

[21] Gee A, McGarty C, Banfield M (2016). Barriers to genuine consumer and carer participation from the perspectives of Australian systemic mental health advocates. J Ment Health.

[22] Knoblock-Hahn AL, Wray R, LeRouge CM (2016). Perceptions of adolescents with overweight and obesity for the development of user-centered design self-management tools within the context of the chronic care model: a qualitative study. J Acad Nutr Diet.

[23] Lyles CR, Sarkar U, Osborn CY (2014). Getting a technology-based diabetes intervention ready for prime time: a review of usability testing studies. Curr Diab Rep.

[24] LeRouge C, Ma J, Sneha S, Tolle K (2013). User profiles and personas in the design and development of consumer health technologies. Int J Med Inform.

[25] Wagner EH, Austin BT, von Korff M (1996). Organizing care for patients with chronic illness. Milbank Q.

[26] Schmittdiel JA, Shortell SM, Rundall TG, Bodenheimer T, Selby JV (2006). Effect of primary health care orientation on chronic care management. Ann Fam Med.

[27] Wagner EH, Glasgow RE, Davis C, Bonomi AE, Provost L, McCulloch D, Carver P, Sixta C (2001). Quality improvement in chronic illness care: a collaborative approach. Jt Comm J Qual Improv.

[28] Wagner EH, Austin BT, Davis C, Hindmarsh M, Schaefer J, Bonomi A (2001). Improving chronic illness care: translating evidence into action. Health Aff (Millwood).

[29] Stellefson M, Chaney B, Barry AE, Chavarria E, Tennant B, Walsh-Childers K, Sriram P, Zagora J (2013). Web 2.0 chronic disease self-management for older adults: a systematic review. J Med Internet Res.

[30] Dietz WH, Solomon LS, Pronk N, Ziegenhorn SK, Standish M, Longjohn MM, Fukuzawa DD, Eneli IU, Loy L, Muth ND, Sanchez EJ, Bogard J, Bradley DW (2015). An integrated framework for the prevention and treatment of obesity and its related chronic diseases. Health Aff (Millwood).

[31] Dietz W, Lee J, Wechsler H, Malepati S, Sherry B (2007). Health plans' role in preventing overweight in children and adolescents. Health Aff (Millwood).

[32] Venkatesh V, Morris MG, Davis GD, Davis FB (2003). User acceptance of information technology: toward a unified view. MIS Q.

[33] Venkatesh V, Thong JY, Xu X (2012). Consumer acceptance and use of information technology: extending the unified theory of acceptance and use of technology. MIS Q.

[34] Tavares J, Oliveira T (2016). Electronic health record patient portal adoption by health care consumers: an acceptance model and survey. J Med Internet Res.

[35] Henneman L, Timmermans DR, van der Wal G (2006). Public attitudes toward genetic testing: perceived benefits and objections. Genet Test.

[36] Hoque R, Sorwar G (2017). Understanding factors influencing the adoption of mHealth by the elderly: an extension of the UTAUT model. Int J Med Inform.

[37] Cimperman M, Brenčič MM, Trkman P (2016). Analyzing older users' home telehealth services acceptance behavior-applying an extended UTAUT model. Int J Med Inform.

[38] Baumeister H, Nowoczin L, Lin J, Seifferth H, Seufert J, Laubner K, Ebert D (2014). Impact of an acceptance facilitating intervention on diabetes patients' acceptance of internet-based interventions for depression: a randomized controlled trial. Diabetes Res Clin Pract.

[39] Gallant MP (2003). The influence of social support on chronic illness self-management: a review and directions for research. Health Educ Behav.

[40] Schwartz LA, Daniel LC, Brumley LD, Barakat LP, Wesley KM, Tuchman LK (2014). Measures of readiness to transition to adult health care for youth with chronic physical health conditions: a systematic review and recommendations for measurement testing and development. J Pediatr Psychol.

[41] LeRouge C, Durneva P, Sangameswaran S, Gloster A (2019). Design guidelines for a technology-enabled nutrition education program to support overweight and obese adolescents: qualitative user-centered design study. J Med Internet Res.

[42] Sandelowski M (1995). Sample size in qualitative research. Res Nurs Health.

[43] Faulkner L (2003). Beyond the five-user assumption: benefits of increased sample sizes in usability testing. Behav Res Methods Instrum Comput.

[44] Glaser B, Strauss A (1967). The Discovery of Grounded Theory.

[45] Mason M (2019). Sample size and saturation in PhD studies using qualitative interviews. Qual Soc Res.

[46] Morgan D (1997). Focus Groups as Qualitative Research.

[47] Krueger R, Casey M (2009). Planning the focus group study. Focus Groups: a Practical Guide for Applied Research.

[48] Morse JM (1995). The significance of saturation. Qual Health Res.

[49] Lee A, Baskerville R (2003). Generalizing generalizability in information systems research. Inf Sys Res.

[50] Saldaña J (2013). The Coding Manual for Qualitative Researchers.

[51] LeRouge C, Dickhut K, Lisetti C, Sangameswaran S, Malasanos T (2016). Engaging adolescents in a computer-based weight management program: avatars and virtual coaches could help. J Am Med Inform Assoc.

[52] Jiang H, Li M, Wen LM, Baur L, He G, Ma X, Qian X (2019). A community-based short message service intervention to improve mothers' feeding practices for obesity prevention: quasi-experimental study. JMIR Mhealth Uhealth.

[53] Holtz BE, Murray KM, Hershey DD, Dunneback JK, Cotten SR, Holmstrom AJ, Vyas A, Kaiser MK, Wood MA (2017). Developing a patient-centered mhealth app: a tool for adolescents with type 1 diabetes and their parents. JMIR Mhealth Uhealth.

[54] Waite-Jones JM, Majeed-Ariss R, Smith J, Stones SR, Van Rooyen V, Swallow V (2018). Young people's, parents', and professionals' views on required components of mobile apps to support self-management of juvenile arthritis: qualitative study. JMIR Mhealth Uhealth.

[55] Hammersley ML, Jones RA, Okely AD (2016). Parent-focused childhood and adolescent overweight and obesity ehealth interventions: a systematic review and meta-analysis. J Med Internet Res.

[56] Cushing CC, Fedele DA, Brannon EE, Kichline T (2018). Parents' perspectives on the theoretical domains framework elements needed in a pediatric health behavior app: a crowdsourced social validity study. JMIR Mhealth Uhealth.

[57] El-Gayar O, Timsina P, Nawar N, Eid W (2013). Mobile applications for diabetes self-management: status and potential. J Diabetes Sci Technol.

